# Supplementary material for: Flexible and Reusable Ag Coated TiO2 Nanotube Arrays for Highly Sensitive SERS Detection of Formaldehyde
Source: Molecules. 2020 Mar 6;25(5):1199. doi: 10.3390/molecules25051199 (PMC7179449; doi:10.3390/molecules25051199)
Supplement: Supplementary file 1 [file molecules-25-01199-s001.pdf]

*Supplementary Materials*

# Flexible and Reusable Ag Coated $TiO_2$ Nanotube Arrays for Highly Sensitive SERS Detection of Formaldehyde

## Figure S1

It is worth emphasizing that when testing SERS, the silver sol used was a liquid. Due to the limitations of SEM and EDX technology, only the Ag distribution on the TNA surface after the silver sol is dried can be characterized; the drying progress results in some aggregation of Ag nanoparticles. Therefore, this characterization cannot completely show the state of Ag distribution caused by the SERS test. The SERS test can be completed in 1 min; during this time, the Ag nanoparticles are moving, and their distribution is more uniform than that shown in the figure.

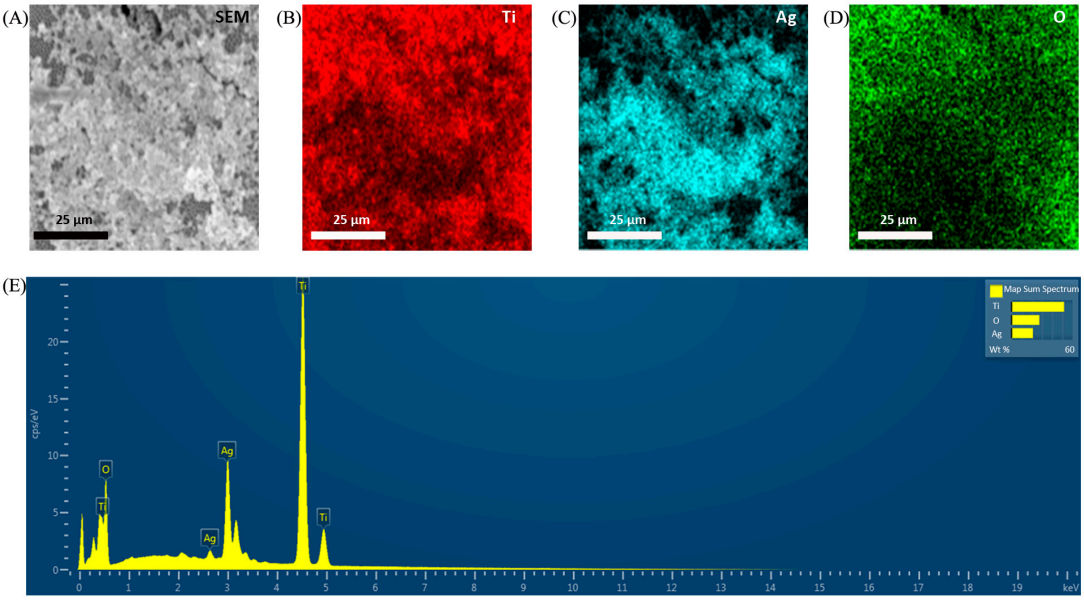


**Figure S1. (A)** Top-view SEM image of the Ag-TNA; **(B–D)** energy dispersive X-ray (EDX) mapping analysis of the Ag-coated TNA material and the mapping distribution of **(B)** Ti, **(C)** Ag, and **(D)** O; **(E)** the EDX spectrum of the Ag-coated TNA material.

## Figure S2

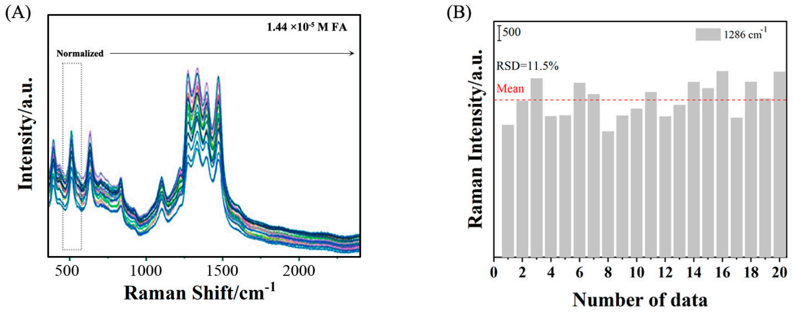


**Figure S2.** Repeatability and reproducibility measurement of the detection of FA obtained on 20 spots randomly selected on the Ag-TNA substrates. (**A**) SERS spectra of 1.44 × $10^{-5}$ M FA and (**B**) intensity distribution of the band at 1286 $cm^{-1}$ after normalizing the SERS spectra of (**A**) to the 514 $cm^{-1}$ band ($A_{1g}$/$B_{1g}$ mode of anatase $TiO_2$). The average intensity is indicated with a red dashed line.
